# Protective or Detrimental? Understanding the Role of Host Immunity in Leishmaniasis

**DOI:** 10.3390/microorganisms7120695

**Published:** 2019-12-13

**Authors:** Camila dos Santos Meira, Lashitew Gedamu

**Affiliations:** Department of Biological Sciences, University of Calgary, Calgary, AB T2N 1N4, Canada; lgedamu@ucalgary.ca

**Keywords:** *Leishmania*, immunopathology, cutaneous leishmaniasis, visceral leishmaniasis, double-stranded RNA virus (LRV), sandfly, innate immunity, adaptive immunity

## Abstract

The intracellular protozoan parasites of the genus *Leishmania* are the causative agents of leishmaniasis, a vector-borne disease of major public health concern, estimated to affect 12 million people worldwide. The clinical manifestations of leishmaniasis are highly variable and can range from self-healing localized cutaneous lesions to life-threatening disseminated visceral disease. Once introduced into the skin by infected sandflies, *Leishmania* parasites interact with a variety of immune cells, such as neutrophils, monocytes, dendritic cells (DCs), and macrophages. The resolution of infection requires a finely tuned interplay between innate and adaptive immune cells, culminating with the activation of microbicidal functions and parasite clearance within host cells. However, several factors derived from the host, insect vector, and *Leishmania* spp., including the presence of a double-stranded RNA virus (LRV), can modulate the host immunity and influence the disease outcome. In this review, we discuss the immune mechanisms underlying the main forms of leishmaniasis, some of the factors involved with the establishment of infection and disease severity, and potential approaches for vaccine and drug development focused on host immunity.

## 1. Introduction

*Leishmania* is the genus of more than 20 digenetic protozoan parasites from the Trypanosomatidae family that causes the vector-borne diseases collectively known as leishmaniasis, a serious public health problem with estimated 0.7–1 million new cases per year [1,2,3]. Leishmaniasis is considered a chronic disease of marginalized communities, and it has been largely neglected in spite of being classified by the World Health Organization (WHO) as one of the most common parasitic infections in the world [2,4]. A broad spectrum of clinical manifestations is attributed to leishmaniasis, which can range from self-healing localized cutaneous lesions to life-threatening visceral disease. The type and severity of the clinical manifestations are mostly determined by the infecting *Leishmania* spp. However, other factors, such as vector biology and host immune status, greatly influence the disease outcome [5].

*Leishmania* are obligate intracellular parasites with a digenetic life cycle. The flagellated promastigotes reside and multiply in the midgut of sandflies (genus *Lutzomya* in the New World and *Phlebotomous* in the Old World), and they are transmitted to mammalian hosts by infected female sandflies during blood feeding [6]. Once in a mammalian host, promastigotes rapidly differentiate into non-motile round-shaped amastigotes within mononuclear phagocytes, where they proliferate and establish infection in phagosomes [6,7].

The clearance of these intracellular parasites and, ultimately, the infection resolution involves the coordinated participation of both innate and adaptive immunity, a process that demands precise regulation. However, suppression or exacerbation of the immune responses promotes the characteristic immunopathology of leishmaniasis [8]. Here, we will review the immune mechanisms driving the diverse clinical forms of leishmaniasis, focusing on the factors that contribute to disease severity and potential approaches to circumvent these limitations.

## 2. Clinical Aspects of Leishmaniasis

The clinical manifestations of leishmaniasis are diverse and multifactorial, largely depending on the infecting species, host immunological status, and other factors related to the vector and the environment [8]. The main forms of the disease are traditionally classified according to their symptomatology—cutaneous leishmaniasis (CL) is the most prevalent, while visceral leishmaniasis (VL) is the most severe form [2] (Table 1).

Cutaneous leishmaniasis predominantly affects the skin of infected individuals and can manifest as localized CL (LCL), diffuse CL (DCL), and mucocutaneous leishmaniasis (MCL) [7]. LCL is the most common manifestation, responsible for up to 95% of all CL cases. It results in a single or small number of lesions at the vector’s biting sites that may gradually progress within weeks or months, from papules/nodules to ulcerated lesions [9]. In sporadic cases, multiple lesions may occur on the body, which is considered a type of disseminated cutaneous disease [10]. Typically, cutaneous lesions resolve spontaneously upon the efficacious establishment of the cell-mediated immune response [11]. Two LCL etiological agents are *L. amazonensis*, in New World, and *L. major*, in the Old World, with particular clinical features being attributed to each of them [2].

DCL is an atypical CL form and occurs when a defective cell-mediated immune response against *Leishmania* parasites gives rise to disseminated, nodular, non-ulcerating, and non-healing cutaneous lesions that affect the entire body with intense parasite proliferation [11]. DCL patients are often refractory to treatment, and DCL cases have been reported in South and Central America, Kenya, and Ethiopia, mainly caused by *L. amazonensis*, *L. mexicana,* and *L. aethiopica* [10].

MCL accounts for 1–10% of CL cases in endemic areas [7]. This form of leishmaniasis is characterized by an exacerbated cell-mediated immunity, which, despite controlling parasite proliferation, also promote intense inflammation and tissue destruction [12]. Patients treated for LCL can manifest MCL later, after apparent resolution of primary lesions. In MCL, parasites metastasize to mucosal tissues of the upper respiratory tract (e.g., naso-oral and pharyngeal cavities), causing an erosive disease that leads to disfiguring lesions and facial mutilations [5]. More than 90% of MCL cases have been reported in three South American countries (Brazil, Peru, and Bolivia) and, although other *Leishmania spp*. are associated with this manifestation, such as *L. major*, *L. panamensis*, *L. tropica,* and *L. infantum*, MCL has been commonly observed in *L. braziliensis* infections [2,5].

Visceral leishmaniasis (also known as “Kala-azar”) is the most serious form of leishmaniasis and predominantly fatal when patients do not receive proper treatment [10]. VL targets internal organs, such as liver and spleen, after parasite dissemination, compromising the reticuloendothelial system [13]. VL patients may initially develop an asymptomatic infection, which escalates to a systemic condition involving splenomegaly, hepatomegaly, weight loss, persistent fever, anemia, among other syndromes [14]. Infected individuals display high levels of antibodies and intense parasite expansion in the targeted organs, including bone marrow [14]. In 5–15% of VL cases, treated patients develop a chronic form of CL, called Post-Kala-azar Dermal Leishmaniasis (PKDL), with the appearance of non-ulcerating cutaneous lesions [15]. VL is typically caused by *L. donovani* and *L. infantum* strains in the Old World and New World, respectively, and it is frequently reported in countries such as India, Nepal, Bangladesh, Sudan, and Brazil [15,16].

## 3. The Immunobiology of Leishmaniasis

The cellular and immunological mechanisms associated with *Leishmania* infection are not completely clear, and most of the current knowledge on this subject is primarily based on experimental models of leishmaniasis. The well-established animal models for leishmaniasis have been shown to reproduce some immunopathological aspects of the human disease, though with limitations, allowing the investigation and characterization of regulatory factors linked to resistance or susceptibility to *Leishmania* and their implications to the infected host [17]. Although substantial differences are observed between experimental and human leishmaniasis, the crosstalk between innate and adaptive immune components and the type of the cell-mediated responses elicited are critical factors determining the fate of the disease in both cases [14,17,18].

### 3.1. Early Events

Once inoculated into the host dermis by an infected sandfly, infective metacyclic promastigotes of *Leishmania* are engulfed by a variety of immune cells, such as resident dermal dendritic cells, macrophages and infiltrating neutrophils that act as the first line of defense [20,21,22,23,24]. The rapid and massive recruitment of neutrophils to the site of parasite inoculation has been well documented and associated with the enhancement of the disease [20,21,22]. Neutrophil-driven inflammation seems to be mostly triggered by the insertion of the sandfly’s proboscis into the skin, provoking tissue damage [24,25]. Other vector-derived components that appear to participate in the recruitment of neutrophils is its saliva, which contains anti-hemostatic mediators (vasodilators and anticoagulants) and immunomodulatory elements [26], such as adenosine, AMP and nucleotidases, delivered to the host along with parasite-derived components, such as exosomes [27] and proteophosphoglycans, particularly the Promastigote Secretory Gel (PSG) [28]. PSG is a mucin-like gel produced by promastigotes in the sandflies that accumulates in and blocks the vector mouthparts, forcing the infected sandflies to regurgitate several times during blood feeding, a behavior hypothesized to enhance the chances of parasite transmission and inflammation [6,28]. In addition to their contribution to preventing the formation of blood clots, the hydrolysis of ATP by vector-derived nucleotidases followed by increased levels of extracellular AMP and adenosine have been suggested to exacerbate lesions in experimental models of leishmaniasis, most likely through activation of the purinergic receptor A_2A_ on immune cells, leading to inhibition of inflammatory functions, such as monocyte maturation, phagocytosis, and nitric oxide (NO) production [29].

**Figure 1:**
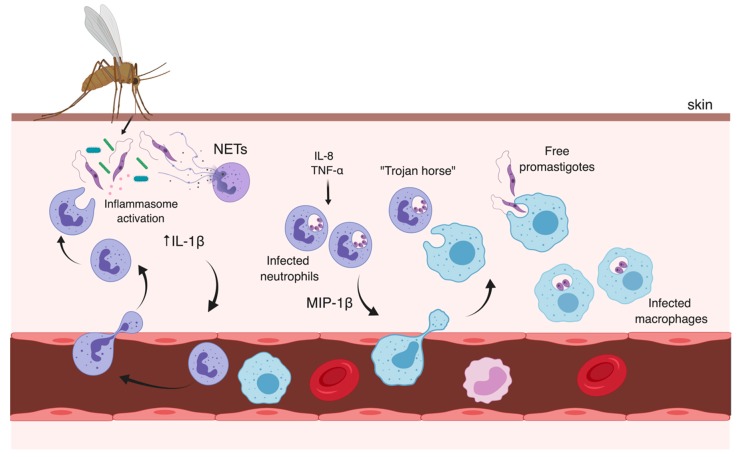
Recruitment of innate immune cells in *Leishmania* infection. Sandfly-derived components, such as anti-hemostatic mediators, adenosine, AMP, and gut microbes, are co-inoculated into the dermis with metacyclic promastigotes and other parasite-derived elements, such as exosomes and Promastigote Secretory Gel (PSG). Rapid and sustained recruitment of neutrophils to the site of inoculation is partially driven by immunomodulatory components of the vector’s saliva. Sandfly’s gut microbiota has been demonstrated to induce the inflammasome activation and release of IL-1β, which promotes inflammation and acts as an autocrine signal, amplifying neutrophil infiltration. Neutrophil extracellular traps (NETs) can capture and kill some parasites, while infected neutrophils degranulate and release several inflammatory mediators, including the chemokine Macrophage Inflammatory Protein (MIP)-1β, stimulating the recruitment of monocytes and macrophages. Apoptotic neutrophils with viable parasites act as “Trojan Horses”, silently transferring amastigotes to macrophages. Free promastigotes escaping from apoptotic cells can also be internalized by macrophages, where they differentiate into amastigotes. Image created with BioRender.com.

An additional explanation for the large and sustained neutrophil infiltration at the bite sites was recently proposed and involves the immunomodulatory properties of gut microbes from infected sandflies that are co-egested with *Leishmania* parasites into the skin (Figure 1). Using a VL BALB/c mouse model intradermally infected in the ears by *L. donovani*, Dey et al. demonstrated that the microbiota of the sandfly midgut enhances the early recruitment of neutrophils and the activation of the inflammasome in these cells, with the production of interleukin (IL)-1β, a potent proinflammatory cytokine [30]. These effects were abrogated with the pre-treatment of sandflies with antibiotic cocktails to decrease the microbial population prior to infection, or when mice were treated with an IL-1 receptor (IL1R) antagonist. The authors concluded that the microbe-mediated IL-1β production serves as an autocrine signal that amplifies neutrophil infiltration at the infection sites and may also aid in parasite dissemination to the spleen [30].

Indeed, other studies have already attested the chemotactic activity of the sandfly saliva for neutrophils and macrophages employing other experimental models, with multiple combinations of *Leishmania*-sandfly species, and this activity is most likely important for early delivery of parasites to neutrophils [25,26]. The uptake of *Leishmania* by neutrophils has been observed as early as 30 min after inoculation, and the majority of parasites were still detected in these cells 18 h after infection [5,21]. However, an in vivo study tracking *L. major* parasites in the dermis using intra-vital two-photon imaging detected parasite proliferation only four days after the sandfly bite and a single parasite was enough to initiate the infectious process [24]. Peters et al. noticed that the antibody-induced depletion of neutrophils in C57BL/6 mice, which develop self-healing cutaneous lesions when infected by *L. major*, greatly reduced the amount of viable *L. major* parasites at the infection site [21]. Interestingly, they also observed an increased release of IL-1α and IL-1β pro-inflammatory cytokines from cells at the infection sites in neutrophil-depleted mice, one week post-infection [21]. Similarly, early uptake of *L. mexicana* by neutrophils was associated with poor parasite clearance in vivo, chronic lesions, and impaired recruitment of inflammatory cells to infection sites, while infected neutropenic or antibody-mediated neutrophil-depleted mice had better control of the disease [31]. The infiltration of neutrophils is also observed in chronic lesions of CL patients, but their contribution to the chronic status of leishmaniasis remains elusive [32,33]. It has been proposed that neutrophils facilitate *Leishmania* infection by having better access to parasites than other phagocytic cells in extracellular spaces and by promoting their safe transition to mononuclear phagocytes [21,22]. In this context, apoptotic neutrophils harboring viable parasites could act as “Trojan Horses”, silently transferring parasites to macrophages upon uptake of these dying neutrophils, avoiding cell activation [22]. Later, a mechanism in which parasites escape apoptotic neutrophils to infect macrophages was also admitted [21]. Conversely, neutrophils have also been shown to play protective roles after recognition and phagocytosis of some *Leishmania* spp. [34]. These cells are able to eliminate microbes in mature lysosomes with granule-associated cytotoxic components and production of reactive oxygen species (ROS), or by induction of NETosis, a cell death mechanism that involves the extracellular release of decondensed chromatin, histones, and multiple microbicidal proteins, forming neutrophil extracellular traps (NETs) that capture and kill several pathogens [35,36]. While NETs from human neutrophils can kill *L. amazonensis* parasites and are also reported in cutaneous lesions of patients with leishmaniasis, they were unable to exert a similar leishmanicidal effect on *L. mexicana*, *L. donovani,* or *L. infantum* parasites [37]. Therefore, the impact of neutrophils on the disease outcome may differ according to the experimental model and the infecting species being investigated, revealing that the contribution of neutrophils to the pathogenesis of *Leishmania* infection is far more complex than initially predicted and requires further investigation.

### 3.2. Later Moments After Infection

Though parasite replication within infected neutrophils was already observed for *L. mexicana* in vitro [38], tissue macrophages and monocytes are the major cellular populations harboring *Leishmania* parasites several days after infection [39,40]. The recruitment of monocytes is partially driven by degranulation of infected neutrophils, which also release the chemokine Macrophage Inflammatory Protein (MIP)-1β, CCL3, and other inflammatory mediators in response to various stimuli, such as IL-8 produced by tissue-resident macrophages, complement factors (e.g., C5a) and Tumor Necrosis Factor (TNF)-α [41,42,43] (Figure 1). Macrophages are the main host cells of *Leishmania* parasites, where internalized promastigotes differentiate into non-motile amastigotes, which undergo robust replication and can persist in phagosomes, promoting latent-infections that can be reactivated [40,44]. However, immature inflammatory monocytes rather than tissue-resident macrophages or dendritic cells (DCs) were shown to act as major facilitators of *L. major* expansion and persistence in vivo during primary infections and parasite internalization seems to delay the maturation of these cells [45]. The engulfment of Leishmania promastigotes is mediated by classical membrane-bound phagocytic receptors such as the complement (CR1 and CR3), mannose/fucose (MR) and fibronectin receptors [40,46,47], and caveolin-dependent endocytosis was recently demonstrated to mediate the internalization of *L. donovani* parasites by host cells [48]. A number of promastigote surface proteins are implicated in the initiation of phagocytosis, including the abundant lipophosphoglycan (LPG), the metalloprotease GP63, and proteophosphoglycans (PPGs), which seem to be targeted by host opsonins, such as complement components (C3b/iC3b), galectins, and mannose-binding protein [49,50,51,52]. Moreover, amastigotes released after cell rupture can be coated by host IgG and captured by other phagocytes through Fc receptors (FcyR), a strategy that seems to greatly influence downstream signaling that benefits the parasites [53].

### 3.3. The Adaptive Immune Responses in Leishmaniasis

**Figure 2:**
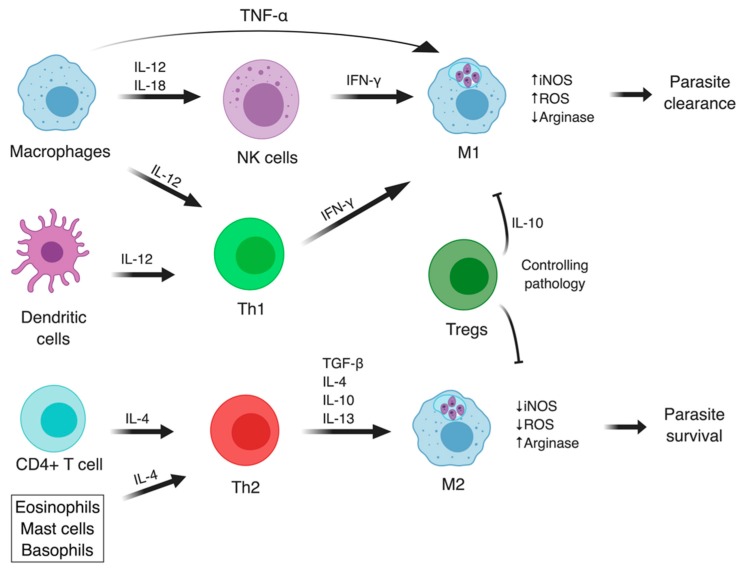
Macrophage polarization. Distinct stimuli induce the M1 or M2-like phenotypes in infected macrophages, which depend on CD4+ T cell differentiation in Th1, Th2, Tregs, among other subsets not shown here. Infected macrophages and dendritic cells (DCs) produce proinflammatory cytokines, such as IL-12, which induce Th1 differentiation. Th1 and Natural Killer (NK) cells, in turn, release interferon (IFN)-γ, stimulating iNOS expression and activity in infected cells, which promote parasite killing. On the other hand, Th2 differentiation leads to production of anti-inflammatory cytokines, which downregulate iNOS activity and stimulates arginase, culminating in parasite survival and proliferation. Tregs promote the balance of pro- and anti-inflammatory responses, avoiding tissue destruction, and controlling pathology (adapted from Maspi et al. [60]). Image created with BioRender.com.

The interaction between *Leishmania* spp. and their host cells will ultimately determine the infection course and the disease outcome of both experimental and human leishmaniasis. The infection resolution is attributed to the establishment of cell-mediated immunity, specifically the activation and differentiation of T lymphocytes that stimulate the production of cytokines, which induce the activation of infected mononuclear phagocytes and culminate with parasite elimination [54]. The differentiation of CD4+ T cells in T helper type (Th)1 upon antigen recognition on Major Histocompatibility Complex (MHC) II is predominantly associated with the development of a proinflammatory response, characterized by secretion of TNF-α, IL-1β, IL-6, IL-12, IL-18, and IL-23 cytokines; increased production of highly microbicidal ROS and reactive nitrogen species (RNS) (e.g., hydrogen peroxide, superoxide, hydroxyl radicals, and NO) via activation of the NADPH oxidase complex and inducible nitric oxide synthase (iNOS), respectively; and enhanced phagocytosis, leading to infection control especially in experimental models [54,55]. Meantime, an anti-inflammatory phenotype is correlated with a predominant Th2 response characterized by the production of IL-4, IL-13, IL-10, and Transforming Growth Factor (TGF)-β cytokines; enhanced arginase activity; polyamine biosynthesis; and IL-21-mediated down-regulation of iNOS, TNF-α, and Toll-like receptor (TLR)-4, favoring intracellular proliferation of *Leishmania* parasites and disease progression [56,57] (Figure 2). Additionally, the role of other T cell subtypes in *Leishmania* pathogenesis, such as T regulatory cells (Treg), CD8+ T cytotoxic, and Th17 effector cells, is coming to light [20,58,59]. Th17 cells are suggested to participate in the balance of inflammatory cytokines, modulating adaptive immunity, and also secret the IL-17 cytokine that contributes to neutrophil recruitment [20].

However, the interplay between innate and adaptive immune systems is far more complex in human leishmaniasis and many other factors influencing the natural infection (e.g., vector and parasite-derived components, concomitant infections with viruses and other microorganisms, host background, etc.), which are not reproduced in experimental models, indeed contribute to the fate of the disease [5,10,25,27,30,61].

#### 3.3.1. Cutaneous Manifestations

**Figure 3:**
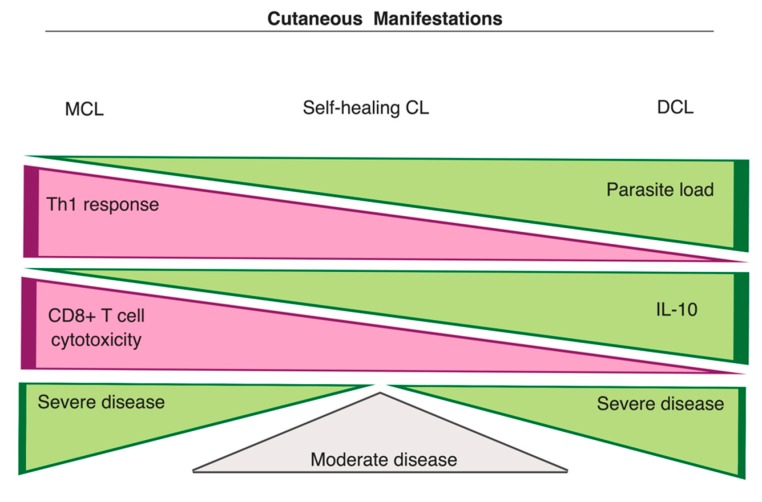
Main features of cutaneous manifestations. This spectrum shows the characteristics of immune responses in MCL (severe disease), CL (moderate disease), and DCL (severe disease). MCL patients develop an exacerbated Th1 response and present high numbers of CD8+ T cells, which promote disease severity. On the other side, DCL is characterized by high parasite loads in lesions, diminished levels of Th1 cytokines, and increased production of IL-10 (adapted from Scott and Novais [11]). Image created with BioRender.com.

The clinical manifestations of leishmaniasis are largely influenced by the amplitude of the host immune responses against the infecting *Leishmania* spp., and these responses can be either protective or pathological. On the one side, patients may develop a strong cellular immunity, controlling parasite proliferation, whereas, on the other side, a response predominantly based on humoral immunity (high levels of anti-leishmanial antibodies) can be mounted, promoting intense parasite proliferation (Figure 3). The balance between these two extremes has been correlated with a moderate disease, where self-healing or chronic lesions are noticed [11,13,14,59].

The immunopathological mechanisms of leishmaniasis have been mainly investigated in mice that reproduce some aspects of the human disease, especially LCL [13]. The infection of C57BL/6 mice with *L. major* is considered a relevant model, since, as in human disease, they develop localized self-healing cutaneous lesions, with low parasite loads, which is associated with activation of DCs and production of IL-12 [21,23]. This cytokine induces differentiation of CD4+ T cells in Th1 effector lymphocytes, which stimulates microbicidal functions of macrophages [62]. However, substantial differences have been observed among CL diseases caused by distinct species, such as *L. mexicana* and *L. amazonensis.* Contrasting with *L. major* infection, *L. mexicana* and *L. amazonensis*, for example, have been shown to survive with a limited Th1 response and the exacerbation of cutaneous lesions caused by *L. amazonensis* infection has been associated with Th1-induced recruitment of cells that enable parasite persistence [63]. Those differences could be partially attributed to the variations in virulence factors observed for each species, such as the surface component LPG, which seems to be crucial in *L. major* infection, but it is not a virulence factor required in *L. mexicana* infection [64] (Box 1). Yet, Th1-related responses seem to be important in controlling *L. amazonensis* proliferation, since IFN-γ-deficient C57BL/6 mice are more susceptible to *L. amazonensis* infection than wild-type mice [65]. In addition, the secretion of IL-1β may also be essential in controlling *L. amazonensis* infection in mice, whereas this cytokine most likely exacerbates disease in *L. major*-infected mice [11]. Actually, the role of IL-1β during *Leishmania* infection is considered controversial [66]. IL-1β is produced as a propeptide that is processed upon assembly and activation of the inflammasome, a molecular complex composed of proteins, such as the NOD-, LRR- and pyrin domain-containing 3 (NRLP3) complex and caspase-1, which mediates IL-1β cleavage [67]. It has been hypothesized that *Leishmania* may activate the inflammasome in the skin indirectly by inducing the production of ROS when parasites are phagocytosed by innate immune cells through C-type lectin receptors [11,67] or by activating a non-canonical pathway, which involves the participation of LPG [66]. Other vector-derived components, including the midgut microbiota co-egested with parasites, may also participate in IL-1β activation [30]. It has been shown that IL-1β can promote IL-12-mediated expansion of Th1 cells, and stimulates NO and TNF production, which contribute to eliminating parasites [60,67]. However, a study with patients infected by *L. mexicana* has associated elevated IL-1β expression with disease severity [68]. Therefore, the mechanisms responsible for resistance or susceptibility also depend on the infecting species that causes CL.

Box 1 **Virulence factors involved in the host immune evasion.**

- Lypophosphoglycan (LPG) and the zinc-metalloprotease GP63: major surface components of *Leishmania* implicated in the impairment of various macrophage functions, including inhibition of phagolysosomal maturation [69,70], cytokine cleavage [71], and activation of negative regulatory factors [72].
- Cathepsin-like cysteine proteases: papain-like cysteine proteases of *Leishmania* were shown to inhibit antigen presentation via major histocompatibility complex (MHC) class II and modulate IL-12 production in macrophages [73,74] and DCs [75]. Cathepsin B-like protease was also implicated in the activation of the latent TGF-β1 in *L. infantum,* and *L. donovani* infected macrophages, inhibiting IFN-γ-induced microbicidal activities [76,77].
- Nucleotidases: both parasite and vector-derived nucleotidases can modulate purinergic signaling mechanisms through increased generation of adenosine, which stimulates the production of IL-10 and inhibits inflammatory functions in neutrophils, DCs, and macrophages [29,78]. Enhanced activity of nucleotidases has been correlated with higher virulence of several *Leishmania* spp. and clinical isolates [79,80].
- Peroxiredoxins (Prxs): components of the unique antioxidant system of trypanosomatids. Prxs work in association with trypanothione, a glutathione analog, to reduce hydrogen peroxide, hydroperoxide, and hydroxyl radicals [81,82,83,84]. Cytosolic Prxs from *Leishmania* were demonstrated to confer protection against peroxides and increased virulence [85,86,87]. Prxs have also been linked to resistance against anti-leishmanial drugs [88].
- Superoxide dismutases (SODs): antioxidant metalloenzymes that convert superoxide to oxygen and hydrogen peroxide. The iron-dependent superoxide dismutase B1 (SODB1) of *L. chagasi* and *L. major* was correlated with parasite proliferation in human macrophages and mice models [89,90], while superoxide dismutase A (SODA) was associated with differentiation and virulence of *L. amazonensis* parasites [91]. The up-regulation of SODA has also been linked to anti-leishmanial drug-resistance (miltefosine) in *L. donovani* infections [92,93].

*L. amazonensis*, *L. mexicana,* and *L. aethiopica* strains are reported as the main etiological agents of DCL, a severe form of CL characterized by the absence of specific cell-mediated response for *Leishmania* antigens, high parasite proliferation and dissemination in humans [8,9] (Figure 3). DCL patients often present with low levels of Th1 cytokines, high antibody titers, and high parasite loads in their lesions [5,94]. The infection of human monocytes with *L. aethiopica* isolated from DCL patients was demonstrated to induce IL-10 expression, which appears to be critical for preventing a protective immunity to *Leishmania*. In contrast, infection with the same species isolated from LCL patients induced higher levels of IFN-γ, IL-6, and IL-4, indicating that distinct immune mechanisms drive the disease outcome of CL subtypes caused by isolates of the same species [95]. Similar observations for IL-10 expression were also made in a recent study with *L. mexicana* strains isolated from LCL and DCL patients [96]. However, the infection of murine bone marrow-derived DCs with DCL-derived *L. mexicana* amastigotes not only promoted higher expression of IL-10 but also of IL-12 and TNF-α [96]. IL-10 seems to play an important role in DCL progression since increased production of this regulatory cytokine is also observed in DCL patients [11]. In fact, it was demonstrated that IL-10 could suppress the IFN-γ-mediated killing of *L. amazonensis* [97]. Other factors that can contribute to parasite proliferation is the higher activation of arginase I and the enhanced production of suppressive TGF-β and prostaglandin E_2_, which are detected in the plasma and skin biopsies of DCL patients [98].

While the full development of a Th1 phenotype, with the involvement of both CD4+ and CD8+ T cells, is critical for the resolution of all forms of CL, the exacerbation of Th1 immune responses leads to extremely severe CL disease [12,59] (Figure 3). The exaggerated cell-mediated immune response is the striking feature of MCL, whereby patients develop secondary metastatic lesions with intense tissue destruction, high levels of proinflammatory cytokines (e.g., IFN-γ) and increased unresponsiveness to anti-inflammatory cytokines, such as TGF-β and IL-10 [12,99]. The attenuated expression of IL-10 receptors (IL-10R), increased levels of TNF-α and the activity of Natural Killer (NK) cells, cytolytic CD8+ T cells and neutrophils also seem to contribute to the clinical outcome of MCL [94,100]. Indeed, patients infected with *L. braziliensis* and presenting with ulcerated lesions have been reported to display higher levels of CD8+ T cells than patients with non-ulcerated lesions [101]. Moreover, IL-17, an inflammatory cytokine involved in neutrophil recruitment, is particularly upregulated in MCL patients [102]. The production of IL-17, which is mostly mediated by Th17 cells, is induced by IL-23 and IL-1β [20]. In turn, IL-1β apparently promotes disease progression in C57BL/6 mice by mediating the expansion of Th17 cells [60]. Curiously, in a study evaluating the transcriptional profiles in cutaneous lesions of *L. braziliensis*-infected patients, higher expression of genes associated with inflammasome pathways was noticed [103]. Thus, considering that *L. braziliensis* infection is often associated with increased chances of clinical complications, including the development of MCL, it is tempting to speculate that IL-1β exerts an influence on the self-feeding inflammation observed in MCL disease by mediating an exaggerated production of inflammatory cytokines and adhesion molecules, increasing recruitment of other cells and amplifying inflammation [11,12].

#### 3.3.2. Leishmania RNA Viruses (LRV) and their Implications in Disease Severity

The presence of an RNA virus in *Leishmania* parasites have been correlated with disease severity and MCL cases both in humans and experimental models. The *Leishmania* double-stranded RNA (dsRNA) viruses (LRV) are cytoplasmic viruses of the family Totiviridae, which include other several viral groups that infect fungi and other parasites, such as *Giardia lamblia* (GLV) and *Trichomonas vaginalis* (TVV) viruses [104]. LRVs have been found in several *Leishmania* spp., including *L. braziliensis*, *L. guyanensis*, *L. major*, and *L. aethiopica* clinical isolates, and phylogenetic analysis attested the existence of at least two divergent LRV groups, LRV1, and LRV2 [100,105]. LRV1 has been predominantly observed in *Leishmania spp*. exclusively found in South America and associated with CL and/or MCL cases, whereas LRV2 has been detected in *L. major* and *L. infantum* isolates [106]. However, a potential new LRV strain (LRV-*Lae*) was more recently characterized from *L. aethiopica* clinical isolates [107].

It was suggested that one of the evolutionary forces possibly driving LRV1 maintenance in these parasites is related to its ability to increase parasite virulence and survival in the human host [108]. LRV1 is considered a potent innate immunogen that can exacerbate disease by stimulating the production of type I interferon (IFN-β) via activation of the Toll-like receptor (TLR)-3 pathway [11,100]. The up-regulation of IFN-β is associated with inhibition of IL-12-mediated DC maturation, diminished T cell-mediated IFN-γ production and down-regulation of IFN-γ receptor (IFNγR), which renders infected cells insensitive to stimuli inducing microbicidal activity, such as NO production [109]. Ives et al. noticed a correlation between infection caused by *L. guyanensis* harboring high levels of LRV1 and disease severity in mice model [110]. Similar to what has been observed for some infections with *T. vaginalis* harboring TVVs [111], infected mammalian cells were able to sense LRV1 dsRNA via endosomal TLR-3, a pathogen recognition receptor, stimulating an increased production of proinflammatory mediators, such as TNF-α and IL-6, known to contribute to the hyper-inflammatory MCL, and anti-viral type I interferons [110]. There is also a chance that viral dsRNA stimulates other receptors, including NOD-like-receptors (NLRs), which could possibly culminate with the activation of the inflammasome and enhance tissue damage [100]. In addition, LRV1-dependent activation of TLR-3 appears to promote the survival of the infected cells through stimulation of the phosphatidylinositol 3-kinase (PI3K)/Akt signaling pathway, involved in cell growth and proliferation [112]. The inoculation of LRV1-infected *L. guyanensis* promastigotes in the footpad of TLR3-deficient mice resulted in reduced swelling and lower parasite loads at the infection site compared to wild-type mice, confirming the involvement of this receptor in disease aggravation. Interestingly, high levels of LRV1 were observed in highly metastasizing *L. guyanensis* strains, which are known to cause MCL [110].

Some studies have indicated a strong correlation between LRV1 in infecting parasites and the development of MCL [113,114,115,116,117]. Ito et al. investigated the presence of LRV1 in MCL patients in the northern part of Brazil and detected the virus in a total of 26 out of 37 cases mainly caused by *L. braziliensis* [113]. Another study evaluating 147 patients from the western part of the Amazon region in Brazil detected a significantly higher incidence of LRV1 in MCL patients (71.1%) than in those with LCL (36.7%), suggesting a higher chance of developing MCL in the presence of LRV1 [114]. A high frequency of LRV1 was also observed in clinical isolates from French Guiana, where LRV1 was detected in, respectively, 55% and 80% of *L. braziliensis* and *L. guyanensis* isolates investigated [115]. Furthermore, treatment failure and disease relapses have been partially attributed to LRV1 in cutaneous infections caused by *L. braziliensis* and *L. guyanensis* [116,117]. However, a cohort study performed with patients from Rio de Janeiro-Brazil revealed that severe manifestations caused by *L. braziliensis* in endemic regions of this state were not correlated with LRV1, implying that additional factors certainly play major roles in disease progression and in the development of MCL, and they probably vary according to geographical location [118].

The mechanisms leading to LRV dsRNA exposure to receptors in mammalian cells are not well understood. Initially, it was speculated that the viral genome could escape from dying parasites in phagolysosomes and then elicit the TLR-3-dependent hyper-inflammation [100]. Recently, a mechanism involving the hijack of the exosomal pathway of *Leishmania* parasites by LRV1 was proposed [119]. LRV1 particles from infected *L. guyanensis* was observed in transmission electron micrographs associated with multivesicular bodies (MVBs) and also reaching the extracellular milieu through exosomes released by its host-parasite [119]. The detection of viral proteins and genome in these membrane-enclosed vesicles by western-blot and RT-PCR indicated that LRV1 uses exosomes as envelopes, which confer protection from viral genome degradation [119]. In addition, LRV1-containing exosomes derived from *L. guyanensis* were able to infect virus-free *L. panamenis* parasites in a transwell migration assay, though the infection only last a few weeks. Importantly, co-inoculation of non-infected *L. panamensis* and *L. mexicana* with LRV1-containing exosomes, but not with naked LRV1, induced disease exacerbation in the mouse model [119]. Hence, the exploitation of exosomes as viral envelopes by LRV1 could possibly explain how the viral genome is exposed to mammalian cells during infection with *Leishmania* and promote hyper-inflammatory reactions. However, the contribution of LRV1-containing exosomes to the immunopathogenesis in the human host remains to be elucidated.

#### 3.3.3. Visceral Manifestations

Compared to CL diseases, the immunopathological mechanisms driving VL manifestations are less understood. However, as for CL, the pathogenesis of VL has been extensively studied in rodent models, especially with *L. donovani* [13]. Yet, contrasting with mice, which mainly develop features of self-healing or subclinical infections, hamsters exhibit disseminated infection, with parasite replication in the spleen, bone marrow, and liver, thus better mimicking, although with limitations, the active human disease [5,13,14]. In experimental models, unique features have been associated with *L. donovani* or *L. infantum* infections [5]. After inoculation into the skin, *L. donovani* rapidly multiplies in macrophages in this area during the first week, with the establishment of granulomas within 4–6 weeks [120]. The initial cutaneous infection seems to be controlled around the 8th week, according to histological observations, but parasites can still disseminate to internal organs, triggering the acute VL disease [120]. In *L. infantum* infections, rapid proliferation of parasites is already observed in the liver during the first four weeks post-infection, nonetheless, as for *L. donovani* infection, parasite growth in spleen seems to be slower, and they can persist in this organ [121]. Hence, it has been hypothesized that the liver is mostly a place for initial parasite replication, while spleen functions as a reservoir of parasites, promoting their persistence [13]. Indeed, it has been demonstrated that splenectomy (spleen removal) appears to minimize the effects of severe VL in humans and, sometimes, allow the successful treatment of refractory cases [122].

**Figure 4:**
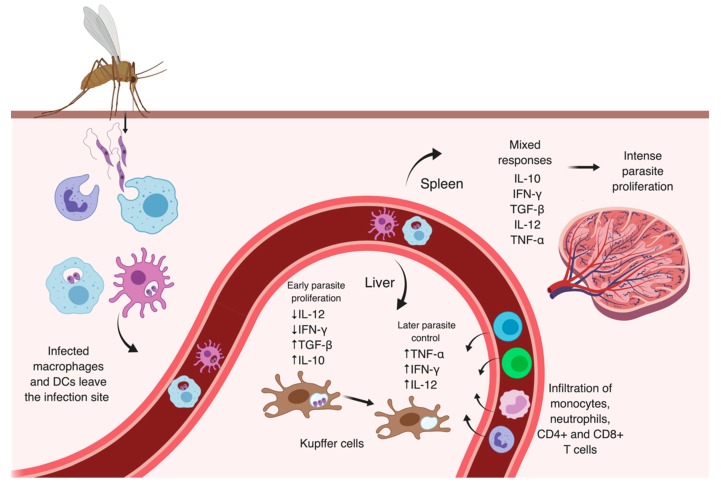
Overview of mechanisms leading to parasite dissemination and proliferation in visceral leishmaniasis. Parasites inoculated into the dermis are captured by innate immune cells. It has been proposed that infected macrophages and dendritic cells (DCs) may leave the infection site and migrate to other areas, disseminating parasites to internal organs, such as liver and spleen. In the liver, early parasite proliferation in Kupffer cells is associated with decreased levels of IL-12 and IFN-γ and up-regulation of anti-inflammatory cytokines. Later, with infiltration of monocytes, neutrophils, CD4+, and CD8+ T cells in liver granulomas, increased levels of proinflammatory cytokines are observed, and infected cells are able to eliminate parasites. However, a heterogeneous pro- and anti-inflammatory responses in the spleen seems to affect the interaction of antigen-presenting cells with T cells and induce intense proliferation of parasites in this lymphoid organ. Image created with BioRender.com.

The factors and determinant mechanisms for parasite visceralization are not clear, but some virulence genes identified in *L. donovani* and *L. infantum*, such as the *A2* gene family, seem to contribute to disease aggravation [123]. *A2* is predominantly expressed during the amastigote stage of *L. donovani* and *L. infantum* and confers resistance to oxidative stress and heat shock [124,125]. In some CL-causing species, such as *L. major* and *L. tropica*, *A2* is a non-expressed pseudogene [123,126]. The expression of *A2* genes in transfected *L. major* parasites induced higher migration of infected cells and increased survival of these parasites in internal organs of BALB/c mice [127]. Interestingly, lower levels of A2 is observed in *L. donovani* causing human PKDL, when parasites move to the skin of the patient after VL treatment [128]. Perhaps, this might explain why viscerotropic parasites are able to better tolerate higher temperatures than cutaneous species [123]. The migration of infected macrophages and DCs to distant sites likely contribute to parasite spread to internal organs, since Zhang et al. observed that higher numbers of DCs and macrophages infected by *L. donovani* leave the intradermal site of parasite inoculation than during *L. major* infection [127] (Figure 4).

Despite the fact that the resolution of VL disease requires both CD8+ and CD4+ T cell participation with IFN-γ and NO production (Th1 response), other immunological factors are involved in this process [5]. It was reported that the disease outcome is primarily associated with specific immune responses generated in the internal organs [13]. In mice liver, *Leishmania* parasites are initially detected in Kupffer cells, specialized resident macrophages, and the main phagocytic population in the liver. Parasites survive in these cells, and their early proliferation seems to be associated with low levels of IFN-γ and IL-12 [14,129]. Later control of parasite growth is observed following the formation of granulomas around infected Kupffer cells in the liver and requires infiltration of blood monocytes, neutrophils, CD8+ and CD4+ T cells, and the production of TNF-α, IFN-γ, and IL-12 that activate infected macrophages to generate ROS and NO that eliminate intracellular amastigotes [5,130] (Figure 4). Using two-photon microscopy, Beattie et al. observed infected Kupffer cells as the only mononuclear population in situ engaging with effector CD8+ T cells for antigen-recognition within *L. donovani*-induced granulomas, suggesting that this interaction could be strategically explored for the development of antigen-based vaccines and immunotherapies [131]. However, mature granulomas are not observed in progressive VL in humans [130].

TGF-β seems to contribute to the inhibition of IFN-γ production in liver granulomas during *L. chagasi* infection, which may lead to parasite survival [132]. Interestingly, increased levels of active TGF-β have been detected in the bone marrow and serum of patients with acute VL, implicating this regulatory cytokine in disease progression [5,133]. Furthermore, it has been noticed that the production of IFN-γ by VL patient-derived peripheral blood mononuclear cells (PBMCs) as well as their proliferation are impaired when they are stimulated with *Leishmania* antigen, contrasting with the responses of PBMCs derived from asymptomatic, cured or subclinical cases [134,135,136]. IL-10 expression has also shown to exert a negative impact on IFN-γ production [13]. This cytokine is considered one of the major suppressors of anti-*Leishmania* responses and high levels of IL-10 is often detected in the serum of VL patients [137]. The high levels of antibodies observed in VL patients are believed to stimulate macrophages to produce IL-10 through the generation of immune complexes that bind to Fc receptors [138]. However, more studies are required to better elucidate the mechanisms inducing IL-10 and TGF-β production and their activity in acute VL disease.

The immune response in the spleen is heterogeneous, and it is possible to observe the production of both Th1 and Th2-related cytokines in spleens of BALB/c mice infected with *L. donovani* parasites, such as IL-10, IFN-γ, TGF-β and IL-12 [13,129] (Figure 4). The progressive infection has been attributed to the redistribution of DCs in this lymphoid organ, which affects the interaction of these cells with T cells and the development of an antigen-specific response. The exacerbated induction of TNF-α and IL-10 are pointed as the major factors contributing to the mislocalization of DCs since they induce downregulation of both CCR7 (also known as CD197) chemotactic receptor on these cells and its ligands, the CCL19 and CCL21 chemokines that are produced by stromal cells in the T-cell rich area [13,14,23]. The impairment of CCL19/CCL21 chemokines in C57BL/6 mice infected with *L. donovani* results in reduced activation and mobility of DCs in the spleen, and elevated levels of IL-10 mRNA that correlates with higher susceptibility to infection [139]. Moreover, the activation of the hypoxia-inducible factor-1α (HIF-1α), typically occurring in poorly oxygenated microenvironments such as inflamed tissues, led to downregulation of IL-12 and upregulation of IL-10 in splenic DCs from an experimental model of chronic VL [140]. In line with these observations, *L. donovani* infection was shown to induce the IRF-5-mediated up-regulation of HIF-1α in DCs also during acute disease, contributing to parasite survival [141]. The expression of HIF-1α in these splenic cells can potentially affect their mobility during *Leishmania* infection since the activation of HIF-1α was demonstrated to alter the expression of the CCR7 chemokine receptor and stimulate the production of IL-1β and TNF-α [139,142]. The cellular relocation in the spleen, with increasing loss of specialized infected macrophages and other cells, leads ultimately to progressive destruction and disruption of the splenic architecture, similar to what is observed in severe VL in humans [14,16]. Therefore, contrasting with its regulated role in resolving the liver infection, the excessive production of TNF-α in spleen along with IL-10 appear to mediate parasite proliferation and chronic infection [130].

PKDL is usually a VL complication observed in some patients after a treatment that generates apparent clinical cure. Thus, PKDL is considered an intermediate state that is preceding the fully VL resolution [15]. This manifestation can appear months or decades after the initial treatment, and it is possibly related to a suppressed immunity towards parasites that persist in the skin, producing skin rashes or non-ulcerating cutaneous lesions with high parasite loads [138]. It has been shown that monocytes and macrophages from these lesions exhibit increased expression of arginase-1, downregulation of TLR-2/4, and decreased production of ROS and RNS, associated with disease chronicity [55]. Interestingly, this type of response in the skin diverges from the predominant Th1 response induced systemically after VL treatment [13]. Keratinocytes producing TGF-β, TNF-α, IL-10, and IL-12 have also been implicated in the development of PKDL [138]. Indeed, elevated IL-10 levels in the plasma and in the keratinocytes of patients have already been proposed to predict PKDL cases in a study in Sudan [143]. Another source of IL-10 is a subset of Treg cells (CD4+CD25+Foxp3+) that were identified in tissue samples of PKDL patients and can also be correlated with TGF-β production [138]. However, other not well-understood responses seem to be involved in PKDL features, which vary according to geographic regions, parasite strains, and immune status of the individuals [15].

## 4. Promising Approaches for Drug Development: A Special Focus on the Host

Conventional treatment of leishmaniasis is based on few chemotherapies associated with toxic side effects, variable efficacy, and drug resistance [5]. Although the main mechanisms underlying the action of antileishmanial drugs are mostly parasitotoxic, these drugs have been shown to negatively affect the host immune system as well [10,144]. In addition, it has been demonstrated that *Leishmania* parasites can evade drug action by remodeling their genetic content, enabling rapid development of resistance to antiparasitic drugs [145]. Thus, therapeutic approaches primarily focusing on “boosting” host immunity against *Leishmania* have been proposed as a better alternative to aggressive treatments currently in use for leishmaniasis that can target host determinants for disease progression [146,147].

De Muylder et al. observed that incubation of *L. donovani*-infected THP-1 macrophages with naloxonazine, a potent antagonist of μ-opioid receptors (MOR), decreased the survival of intracellular parasites. They noticed this effect was associated with increased expression of host vacuolar ATPase (vATPase) transporter, a proton pump recruited to phagolysosomes that mediate the acidification of these compartments. When simultaneously treating infected cells with naloxonazine and a vATPase inhibitor (concanamycin A), the proliferation of parasites was restored [148]. Thus, the remodeling of the host vacuolar system may constitute a potential strategy to control *Leishmania* intracellular growth in acidic compartments.

Using BALB/c mice as experimental VL model for infection with antimony-resistant *L. donovani*, Mukherjee et al. demonstrated the indirectly anti-leishmanial effect of imipramine, an antipsychotic drug used to treat patients with depression [149]. The initial treatment of infected macrophages from BALB/c mice with imipramine was shown to down-regulate IL-10 production and decreased the expression of IL-10-dependent multidrug resistance protein (MDR)-1 pump involved in *L. donovani* resistance to antimonials, the first-line drugs in the treatment of leishmaniasis. The downregulation of IL-10 was associated with imipramine-induced higher expression of host histone deacetylase (HDAC) 11, which impairs the binding of NF-kB p50/Rel-c complex to IL-10 promoter. Interestingly, a shift towards IL-12 production is observed in these cells, which involves the preferential recruitment of NF-kB p65/RelB complex to IL-12 promoters. When treating infected BALB/c mice with the pentavalent antimonial sodium stibogluconate in combination with oral imipramine, they observed a greater reduction of parasite loads in the spleen and the liver of these animals compared to treatment with sodium stibogluconate alone. This effect could be attributed to the IL-12-stimulated restoration of T cell-mediated responses in these organs [149]. Indeed, a study has already noticed that the addition of IL-12 to PBMCs from VL patients restores IFN-γ production, implicating this cytokine in the resolution of VL disease [136].

Another interesting study investigated the antileishmanial activity of imiquimod, a drug classified as a modifier of the immune response, especially acting on monocytes and macrophages, and used, for example, as a topical treatment for genital warts caused by papillomaviruses [150]. The treatment of *L. donovani*-infected bone marrow-derived macrophages (BMM) with imiquimod was shown to control parasite proliferation in these cells. Similarly, when used as a topical treatment on cutaneous lesions of *L. major*-infected BALB/c mice, 5% imiquimod cream induced a significant reduction in the lesions and local parasite loads. Imiquimod did not trigger toxicity directly against parasites, but, instead, it activated infected cells to produce NO. Surprisingly, the authors had evidence for the activation of pathways related to AP-1 and NF-kB in these cells but not for Jak/STAT1 signaling pathway classically associated with IFN-γ induction of iNOS expression [151]. Later, El Hajj et al. demonstrated that the activity of imiquimod and its analog EAPB0503 was mediated by binding and/or up-regulation of Toll-like receptor (TLR)-7, activating the NF-kB pathway. Increased expression of iNOS and production of proinflammatory cytokines, such as IL-12, IL-1β, TNF-α, and IL-6, were also observed after treatment of macrophages infected with distinct CL-causing *Leishmania spp.* in this study [152]. TLRs are an important class of innate pattern recognition receptors, and they have already been implicated in recognition of *Leishmania* parasites (e.g., TLR2, TLR4, and TLR9), mediating the establishment of protective immunity [5]. Therefore, the development of drugs that promote the modulation of TLRs to control intracellular growth and survival of these parasites could be a promising approach.

## 5. Vaccines for Leishmaniasis

Traditional strategies for vaccine development to control leishmaniasis have failed to reproduce the lifelong immunity to reinfection observed in patients clinically recovering from the disease but maintaining chronic infections (concomitant immunity) considered subclinical. This inability to design prophylactic vaccines that trigger long-lasting protection against *Leishmania* antigens in humans reflects the gap in the current understanding of the relationship between disease pathogenesis and host immune responses generated against *Leishmania* parasites, and the challenge of translating experimental evidence from animal models to human cases [18,153].

The inoculation of live and virulent *Leishmania* parasites termed leishmanization was a common vaccination strategy in endemic areas that conferred protection to natural and exacerbated *Leishmania* infections induced by sandfly transmission, but this practice was largely discontinued due to safety and reproducibility issues [154]. Attempts to emulate the anti-*Leishmania* responses acquired by “leishmanized” individuals with whole-killed or attenuated parasite-based formulations have raised concerns regarding the low immunogenicity and the potential reversion to a more virulent phenotype, respectively, in addition to variations in vaccine efficacy observed in clinical trials in different areas [153,154,155]. Yet, the use of adjuvants to tailor immune responses through activation of specific innate pathogen-recognition receptors (PRRs), such as TLRs, has been shown to enhance the generation of Th1 memory cells and parasite-specific responses to secondary challenges [156]. Highly conserved immunogenic proteins or epitopes between *Leishmania* spp. have the potential to confer cross-protection as demonstrated for *L. donovani* nucleoside hydrolase (NH36) and/or its recombinant fragments F1 (N-terminal domain) and F3 (C-terminal domain) formulated with saponin, which induced antigen-specific protection in BALB/c mice against *L. amazonensis* and *L. braziliensis* [157,158,159]. Other *Leishmania* proteins evaluated as vaccine candidates are reviewed in [160].

Similarly, recombinant proteins composed of fused peptides or epitopes have been developed to improve the efficacy of vaccine candidates in heterogeneous populations [160]. To illustrate, the polyprotein Leish-111f (a combination of *L. major* thiol-specific antioxidant [TSA] and stress-inducible protein-1 [LmSTI1], and *L. braziliensis* elongation initiation factor [LeIF]), when adjuvanted with the TLR-4 agonist monophosphoryl lipid A (MPL) + squalene, elicited promising results in trials with healthy and CL patients, and with the recent advances in bioinformatics tools, the identification of more immunogenic combinations of peptides will likely happen [160,161].

An alternative and attractive vaccine approach is the delivery of recombinant nucleic acids (DNA and RNA) encoding *Leishmania* antigenic proteins that can be expressed in vivo and loaded into MHC molecules on transfected cells [162,163]. Some of the advantages of nucleic acid-based vaccines are their stability, the number of antigens and adjuvants that can be encoded by a single vector, the expression of structurally unaltered immunogenic proteins in recipient cells and the antigen-specific stimulation of CD4+ and CD8+ T cell responses [153,164]. Several tests with DNA-based vaccines have been conducted in experimental models of leishmaniasis using different routes of administration. Overall, they seem to confer or enhance protection against a number of *Leishmania* spp. in animal models, but their safety and efficacy in humans remain controversial [162,165,166,167]. However, some candidates were shown to be immunogenic in clinical trials, including an adenoviral-based platform (ChAd63-KH) encoding a polyprotein (a combination of kinetoplastid membrane protein-11 [KMP-11] and hydrophilic acylated surface protein B [HASPB] genes from *L. donovani*), which induced parasite-specific CD8+ T cell responses when tested in Phase I trials, suggesting its potential use as prophylactic and therapeutic vaccine for VL or PKDL [168]. Contrasting with DNA vaccines, mRNAs associated or not with viral vectors have the advantage of accumulating in the cytoplasm and being degraded once proteins are translated, thus minimizing the risks of integration of foreign genetic material into the host genome. Nonetheless, RNA vaccines can be quickly recognized and deteriorated after injection, restricting their availability in the organism [164]. Hence, efforts have been made to improve RNA delivery systems, including the development of more stable formulations with liposomes or nanoparticles [169,170]. A novel and more organic strategy involve the potential use of exosomes to deliver not only nuclei acids but other parasite-derived components, such as lipids and proteins, that can elicit robust immune responses [27,171,172,173,174]. These small extracellular vesicles are naturally employed in cargo transportation among cells, and exosomes loaded with exogenous cargo molecules, such as miRNA and siRNA, have been tested for therapeutic purposes in the cancer field as a less toxic and more efficient alternative for systemic delivery [175].

Recent studies have shown that, in addition to the generation of long-lived memory T cells, effective vaccination strategies should consider other critical factors contributing to the establishment of Th1 concomitant immunity during persistent infection, such as the effect of vector-related components and the subsets of CD4+ T cells mediating this process [176,177]. Using the resistant C57BL/6 mice model chronically infected with *L. major*, Peters et al. demonstrated that a short-lived subset of IFN-γ-producing effector T cells (CD44+CD62L-T-bet+Ly6C+), which require continuous exposure to antigens and are not derived from reactivated memory cells, mediate Th1 concomitant immune response to a secondary infection [178]. Thus, the development of vaccines promoting the maintenance and recruitment of these effector cells to the infection sites could be a successful approach.

Remarkable discrepancies among studies evaluating vaccine efficacy may be caused by differences in the inoculum doses of *Leishmania* parasites, routes of administration, and experimental models of parasite transmission [18,179,180]. Infected sandflies inoculate low doses of parasites into the skin and induce higher inflammatory responses when compared to needle inoculation of parasites in experimental leishmaniasis [26,179]. While vaccination with autoclaved *Leishmania* antigen combined with CpG-ODN adjuvant protected C57BL/6 mice against needle challenge with *L. major*, it failed to protect mice against infected sandfly challenge, implicating the crucial participation of vector-derived factors in the establishment of natural infections [177]. Indeed, sandfly transmission promotes a prolonged influx of neutrophils at the bite sites, which is associated with early modulation of immunological factors that create a favorable microenvironment for parasite growth [21]. Hence, a vaccine that elicits a rapid protective T-cell mediated response and maintains the recruitment of parasite-specific effector cells is likely to prevent the very early establishment of parasites in permissive phagocytes.

## 6. Concluding Remarks

Leishmaniasis is a disease with highly variable and complex manifestations. Although the use of rodent models has allowed the characterization of immunopathological aspects of the disease, they show substantial limitations in reproducing features of human leishmaniasis, especially the immune mechanisms promoting disease severity [5]. Thus, understanding the factors driving the distinct clinical manifestations of leishmaniasis remains a challenge and highlights the complexity and uniqueness involving the relationship of different *Leishmania* spp., and strains with their hosts. Although a clinical cure can be observed in certain cases, the sterile immunity (complete elimination of parasites) is difficult to be achieved and one of the main goals is to develop vaccines that confer long-term protection [17,18]. In addition, the combination of schemes directly targeting parasites and modulating the host immune system is definitely a promising approach and, perhaps, an improved alternative to toxic chemotherapies currently in use [146]. Hence, better knowledge on T cell populations participating in *Leishmania* infection and their interplay with macrophages, monocytes, and DCs, which are also critical in this process [18], will likely lead to novel strategies for the development of efficacious treatments and vaccines.

## Figures and Tables

**Table 1:** Main clinical manifestations of leishmaniasis, corresponding agents, and global distribution.

Leishmaniasis	Most Common Etiological Agents	Manifestations	Geographical Distribution
Cutaneous (CL)	/	/	84% CL cases reported in Afghanistan, Algeria, Brazil, Colombia, Iraq, Pakistan, Peru, the Syrian Arab Republic, Tunisia and Yemen (> 90% MCL cases reported in Brazil, Peru and Bolivia) [5,19]
Localized (LCL)	*L. amazonensis* *L. major* *L. aethiopica* *L. mexicana*	Small nodules or papules at the vector’s bite sites that may progress to ulcerated lesions	
Diffuse (DCL)	*L. amazonensis* *L. mexicana* *L. aethiopica*	Disseminated nodular and non-ulcerating lesions	
Mucocutaneous (MCL)	*L. braziliensis* *L. mexicana* *L. panamensis* *L. major ^1^* *L. infantum ^1^* *L. tropica ^1^*	Metastatic secondary lesions in naso-oral and pharyngeal cavities and tissue destruction	
Visceral (VL)	*L. donovani* *L. infantum* *L. amazonensis ^1^*	Splenomegaly, hepatomegaly, weight loss, persistent fever and anemia. Post-Kala-azar Dermal Leishmaniasis (PKDL): skin rashes or non-ulcerating cutaneous lesions after apparent resolution of VL disease	Most VL cases reported in Brazil, Ethiopia, India, Nepal, Bangladesh, Kenya, Somalia, South Sudan and Sudan [19]

^1^ Presentations usually occurring in immunocompromised patients [5,7].
